# Cardiac magnetic resonance imaging for risk stratification in patient with diabetes mellitus and coronary heart disease - a prospective long-term follow-up study

**DOI:** 10.1186/1532-429X-16-S1-O35

**Published:** 2014-01-16

**Authors:** Dominik Buckert, Vinzenz Hombach, Wolfgang Rottbauer, Peter Bernhardt

**Affiliations:** 1 University of Ulm, Ulm, Germany

## Background

There is evidence that myocardial ischemia in patients with diabetes mellitus is often present without the exhibition of symptoms. Moreover, recent data suggests that silent ischemia is associated with occurrence of cardiac events and poor outcome. Aim of our study was to determine the prognostic value of a reversible perfusion deficit assessed by adenosine perfusion cardiac magnetic resonance imaging (CMR) in a consecutive cohort of diabetes mellitus patients independent of the exhibition of clinical symptoms.

## Methods

Consecutive patients with diabetes mellitus type 2 of any duration that were referred for adenosine perfusion CMR were screened for enrollment. It was irrelevant whether the patients exhibited symptoms that were likely to be of ischemic origin or not. A reversible perfusion deficit was assessed by adenosine-perfusion imaging and late gadolinium enhancement on a 1.5 T whole-body CMR-scanner and was defined as hypoperfusion during adenosine infusion without corresponding late gadolinium enhancement. The primary endpoint was defined as cardiac death, non-fatal myocardial infarction or stroke.

## Results

The study population consisted of 239 consecutive patients with prior diagnosed diabetes mellitus. During the follow-up period of 5.0 ± 2.1 years 33 primary endpoints occurred. Patients with an event were significantly older (67.7 ± 8.6 years vs. 63.6 ± 10.3 years, p = 0.02), had lower left ventricular ejection fractions (53.9 ± 18.2% vs. 60.0 ± 15.3%, p = 0.04) and more often a reversible perfusion deficit (21 (63.6%) vs. 57 (27.7%), p < 0.0001) than patients without. In a multivariate analysis, a reversible perfusion deficit was the strongest predictor for an event with a 3.76-fold increased risk (p = 0.0003). Whether the patients presented symptomatically at enrollment had no influence on their particular risk and event-free survival.

## Conclusions

Adenosine perfusion CMR allows excellent risk stratification in patients with diabetes mellitus, independent of other risk factors or the presence of ischemic symptoms.

## Funding

None.

